# Exploring client satisfaction and determinants of family planning services at public health facilities in Debre Tabor town, Northwest Ethiopia: a mixed-method study

**DOI:** 10.3389/frph.2025.1558606

**Published:** 2025-08-29

**Authors:** Tseganesh Asefa, Winta Tesfaye, Gedamnesh Bitew, Hiwot Tezera Endale

**Affiliations:** ^1^ Department of Medical Nursing, School of Nursing, College of Medicine and Health Science, University of Gondar, Gondar, Ethiopia; ^2^ Department of Physiology, School of Medicine, College of Medicine and Health Science, University of Gondar, Gondar, Ethiopia; ^3^ Department of Epidemiology and Biostatistics, School of Medicine, College of Medicine and Health Science, Injibara University, Injibara, Ethiopia; ^4^ Department of Biochemistry, School of Medicine, College of Medicine and Health Science, University of Gondar, Gondar, Ethiopia

**Keywords:** client satisfaction, determinants, family planning services, public health facilities, Northwest Ethiopia, reproductive health

## Abstract

**Introduction:**

Client satisfaction is a crucial indicator of family planning service utilization, and understanding these insights is essential for designing responsive, client-centered interventions to address barriers to contraceptive use. This study aimed to explore client satisfaction and determinants of family planning services in public health facilities in Debre Tabor town, northwest Ethiopia.

**Methods:**

A convergent parallel mixed-methods design, incorporating both quantitative (facility-based cross-sectional) and qualitative (phenomenological) study approaches, was conducted at public health facilities in Debre Tabor town from April 15 to May 29, 2024. Data were collected using an interviewer-administered questionnaire following a multi-stage stratified random sampling technique. Simultaneously, qualitative data were collected from purposefully selected individuals using an in-depth interview guide. The study used EpiData version 4.6 for data input and SPSS version 25 for logistic regression analysis, using a *p*-value < 0.05 as the cutoff point. After an in-depth interview, the record and field notes were transcribed, translated, and analyzed using a thematic analysis technique.

**Result:**

Out of 379 participants, 62.5% of clients were satisfied with the family planning services. Factors significantly associated with clients' satisfaction were urban residents (AOR = 1.68, *p* = 0.022), government employees (AOR = 0.40, *p* = 0.020), shorter waiting times (AOR = 2.93, *p* = 0.039), method counseling (AOR = 2.32, *p* = 0.005), and receiving the preferred method (AOR = 2.59, *p* = 0.024). From the in-depth interview of 15 participants, two themes emerged: “limited provider-client counseling interaction,” with subthemes of “cultural sensitivity gaps,” “misconceptions,” and “fear of ineffectiveness,” and “pathways to satisfaction,” with subthemes of “dignified care” and “accessibility.”

**Conclusion and recommendations:**

The study found moderate client satisfaction with family planning services in public health facilities, with significant disparities in residence, occupation, waiting time, method counseling, and preferred methods. Clients reported a lack of cultural sensitivity, misconceptions, and a lack of involvement in decision-making. To improve satisfaction, health facilities should strengthen client-centered counseling, address cultural misconceptions, and provide flexible service hours and training for providers.

## Introduction

An estimated 35 million couples worldwide, along with 222 million women in low- and middle-income countries, continue to face challenges in accessing modern family planning services; additionally, 150 million married women desire to delay or avoid childbearing despite unmet needs (1–3). The global challenges are reflected in Ethiopia, where the family planning services were initiated four decades ago and set a strategic goal through the Health Sector Transformation Plan to raise the contraceptive prevalence rate (CPR) to 55% and reduce unmet needs to 10% by 2020; the country reported a CPR of only 27% and unmet needs of 13% in that year (4–7). Even when physical access to family planning services is limited and coverage targets are unmet, the quality of care remains crucial in determining whether available services are effectively utilized and sustained. High service quality fosters greater client satisfaction and builds long-term trust, which are essential for continued engagement with family planning programs (8–10).

Client satisfaction is a crucial indicator of family planning service utilization, as it reflects service quality and influences continued use of contraceptive methods. Evidence suggests that satisfied customers are most likely to maintain usage, adhere to follow-up visits, and recommend others (11–13). Several studies have identified various factors that affect client satisfaction with family planning services, including socio-demographic characteristics, service-related factors, and the nature of provider-client interactions, particularly about respect, privacy, and the adequacy of information provided during counseling. Client satisfaction is also influenced by knowledge, attitudes, and widespread myths and misconceptions (14–17).

While physical access and service availability remain important, they alone are not sufficient to ensure optimal uptake and continued use of family planning services. In this regard, client satisfaction serves not only as an outcome measure but also as a critical feedback mechanism for identifying service delivery gaps and tailoring improvements. Moreover, most existing studies on family planning satisfaction have relied predominantly on quantitative methods. Understanding these deeper insights is essential to designing responsive, client-centered interventions that can address barriers to contraceptive use and reduce unmet needs. Given these considerations, this study aimed to explore client satisfaction and determinants of family planning services in public health facilities in Debre Tabor town, northwest of Ethiopia.

## Methods and materials

### Study design, setting, and period

The study employed a convergent parallel mixed-methods design that combined both quantitative (facility-based cross-sectional) and qualitative (phenomenological) study approaches and was conducted from April 15 to May 29, 2024, at public health facilities in Debre Tabor Town, Northwest Ethiopia. Debre Tabor Town administration is one of the 13 districts and five town administrations found in the South Gondar administrative zone in Ethiopia. It is located 105 km away from Bahir Dar and 666 km away from Addis Ababa. The town has a population of 83,082, with 39,781 men and 43,301 females. The total number of households in the town is 13,200, and the town has one referral hospital, known as Debre Tabor Comprehensive Specialized Hospital (DTCSH), and three health centers, namely Debre Tabor Health Center, Leul Alemayehu Health Center, and Atse Seife Areid Health Center. These public health institutes are currently providing a variety of health services, including family planning, to Debre Tabor residents and others in the community (18).

### Study population

All females of reproductive age who visited family planning services at Debre Tabor town public health facilities during the data collection period were included in the study. The health facility staff who attended the family planning service during the study were excluded from the study.

### Sample size determination and sampling procedure

For the quantitative part of the study, the sample size was determined by using the single population proportion formula by considering the following statistical assumptions: 95% confidence interval (CI), 66.1% proportion [from a previous Ethiopian study at Bahir Dar City, Public Health facilities (19)].

$n\;=\;\frac{{\left(Za/2\right)}^{2}(p)(1\text{-}p)}{{\left(d\right)}^{2}}$, Where, *n* = initial sample size; Z = 1.96, the corresponding Z-score for the 95% CI; *P* = proportion 66.1% = 0.661; d = margin of error = 5% = 0.05.

$$n=\frac{\left(1.96)2\;\times \;0.661(1-0.661\right)}{(0.05)2}=344.$$

By taking a 10% non-response rate, the final sample size was 379.

The study employed a multi-stage stratified random sampling technique to ensure proportional representation of client satisfaction among four public health facilities in Debre Tabor Town: three health centers and one general hospital.

In the first stage, the participants were stratified by health facility on the assumption of relative homogeneity among the clients within each facility. In the second stage, proportional allocation was used to determine the number of participants per facility based on the monthly client flow for family planning services. Out of a total of 4,224 clients, the calculated sample size of 379 was proportionally distributed as follows: DTCSH (*N* = 1,540): *n* = 139; Debre Tabor Health Center (*N* = 1,100): *n* = 99; Leul Alemayehu Health Center (*N* = 880): *n* = 78; Atse Seife Areid Health Center (*N* = 704): *n* = 63.

**Figure 1:**
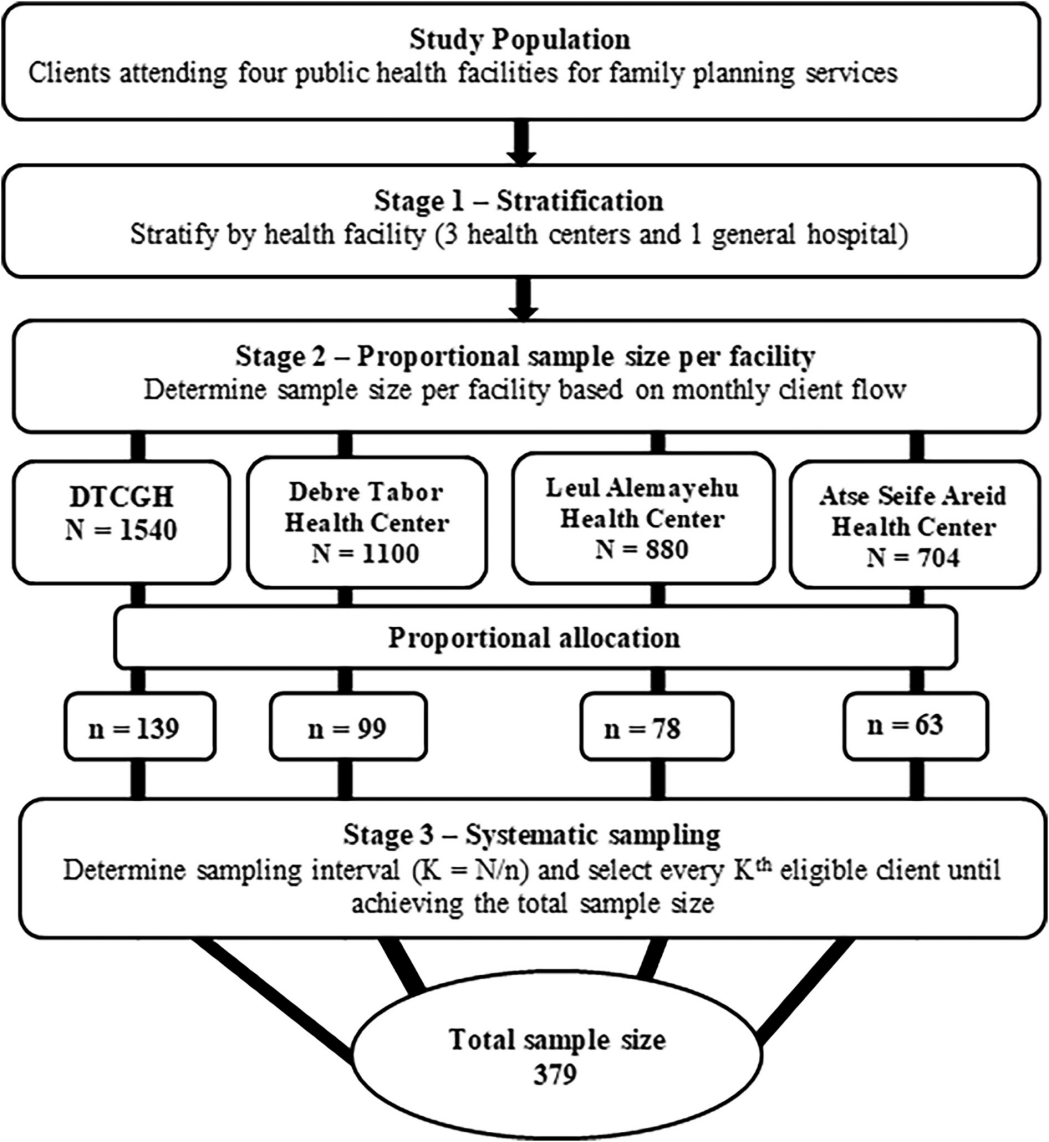
Schematic presentation of the multistage sampling procedure for client satisfaction with family planning services in public health facilities of Debre Tabor Town, Northwest Ethiopia.

In the third stage, a systematic sampling was conducted within each facility. The sampling interval (k) was calculated using the formula $k=\frac{N}{n}$, where N is the number of monthly clients that attend the facility and *n* is the sample size allocated. For all facilities, the interval was approximately 11; every 11th client was selected until the required sample size was obtained. To ensure randomness, the first participant was selected using a simple random sampling method from within the first K individuals, and every K^th^ eligible client was subsequently selected until the required sample size was achieved (Figure 1).

The qualitative study utilized a non-probability purposive sample technique to choose women of reproductive age who were currently using family planning services at four of the public health facilities in Debre Tabor town. Of the total participants, 40% were recruited from the general hospital and the remaining from the health centers, which share similar service characteristics. Efforts were made to ensure variation in age, educational background, and occupation to maximize a range of opinions. Data saturation was achieved at the 13th interview, and two additional interviews were conducted to ensure that no new insights were gained, resulting in a final sample of 15 participants.

### Operational definition

Satisfaction: Clients were considered satisfied if their total satisfaction score, computed by adding replies to all five-point Likert scale items, was more than or equal to the computed overall sample mean. Scores below the mean were considered dissatisfied (19).

### Data collection procedures and quality control

For the quantitative part of the study, data were collected using an Amharic version of a standardized interviewer-administered questionnaire, which was developed from various literature (20–23). The questionnaire assessed family planning satisfaction level using socio-demographic parameters of clients, organizations, and service providers, as well as five-point Likert-scaled items (very dissatisfied = 1, dissatisfied = 2, neutral = 3, satisfied = 4, and very satisfied = 5) to assess family planning satisfaction status. Four data collectors and two supervisors facilitated the data collection process by attending a two-day training session before the study on the study's objectives and ethical issues encountered during the data collection process.

After completing the training, a 5% pre-test was done at Maraki Health Center. During data collection, supervisors reviewed the completeness, accuracy, and consistency of the data. To ensure dependability, it provides important information about data collection, analysis, and interpretation. Further, the data were tested statistically for internal consistency (reliability) using Cronbach's alpha test. The Cronbach's alpha value was 0.76.

For the qualitative portion of the study, an Amharic version of the semi-structured interview guide was utilized to conduct in-depth interviews with women of reproductive age who had received family planning services. The interview guide was developed based on a thorough review of relevant literature and existing tools (24–27). It included open-ended questions and probing questions to explore participants' experiences with service delivery, provider interactions, privacy, cultural considerations, challenges faced, misconceptions, and the perceived impact of services on their satisfaction. Probing questions were used to elicit deeper insights into sensitive or complex topics. The interview was held in a private, quiet room and lasted approximately 40 minutes. Field notes were taken, and the sessions were audio-recorded as part of the data collection process. To ensure trustworthiness, the study adhered to key principles such as credibility, transferability, dependability, and confirmability. Credibility was achieved through prolonged engagement with participants and member checking to validate findings. Field notes and recordings were cross-checked for accuracy to enhance dependability, while confirmability was ensured by maintaining a clear audit trail of the data collection and analysis processes. The researcher carefully reviewed all of the documents.

### Data processing and analysis

For the quantitative part of the study, the collected data were entered using EpiData version 4.6 (28) and analyzed with SPSS version 25 software (29). Descriptive statistical methods were applied to determine frequencies, percentages, and means for both independent and dependent variables. The characteristics of the participants were presented via tables and figures. Associations between variables were assessed using bivariate and multivariate analyses, with statistical significance set at a *p*-value of ≤0.05.

The qualitative data were analyzed using thematic analysis, following an inductive approach. The audio recordings were transcribed and translated from Amharic to English by a bilingual language expert. Field notes were incorporated to enrich the depth and accuracy of the analysis. Two researchers independently conducted manual coding and compared their results for consistency using investigator triangulation. Discrepancies were discussed and resolved through consensus, and a codebook was refined and finalized. These codes were organized into subthemes and themes, which were refined to capture the participants' experiences and viewpoints. Supporting quotes were included to enhance and illustrate the findings. Throughout the code and theme development process, an audit trail was kept to record analytical choices and improve transparency.

## Results

### Socio-demographic characteristics of quantitative study participants

A total of 379 family planning service users were approached for an interview. The mean (±SD) age of respondents was 28.6 (±7.8) years. Of the respondents, 172 (45.4%) were aged 35 years or older, and 284 (74.9%) were married. The majority, 248 (65.4%), of family planning users were Orthodox Christian by religion. Regarding educational status, 130 (34.3%) of respondents had no formal education, while the majority, 249 (65.7%), had formal education. The majority of respondents, 329 (86.8%), were urban residents. Among the participants, 135 (35.6%) were housewives, and 203 (53.6%) reported at least 72,000 ETB per year (Table 1).

**Table 1:** Socio-demographic characteristics of quantitative study participants (*n* = 379).

Variable	Category	Frequency	Percent (%)
Age in years	15–19	52	13.7
	20–24	84	22.2
	30–34	71	18.7
	≥35	172	45.4
	mean (±SD)	28.6 (±7.8) years	
Religion	Orthodox	248	65.4
	Muslim	116	30.6
	Protestant	11	2.9
	Other	4	1.1
Educational status	Cannot write or read	40	10.6
	Read and write	90	23.7
	Primary school	98	25.9
	Secondary school	91	24
	College and above	60	15.8
Marital status	Single	61	16.6
	Married	284	74.9
	Divorced	29	7.6
	Widowed	5	1.3
Occupation	Housewife	135	35.6
	Government employee	60	15.8
	Private employee	54	14.3
	Merchant	68	17.9
	Others	62	16.4
Average annual income	≤72,000 ETB	203	53.6
	>72,000 ETB	176	46.4
Residence	Urban	329	86.8
	Rural	50	13.2

### Organization and service provider-related factors

The majority of the clients visited DTCSH 139 (36.7%), followed by Debre Tabor Health Center 99 (26.3%), Leul Alemayehu Health Center 78 (20.6%), and Atse Seife Areid Health Center 63 (16.4%). A significant portion of the respondents, 293 (77.3%), reported that the opening hours of the family planning room were convenient. Most respondents, 281 (74.1%), were repeat visitors to the family planning clinic, and the majority, 183 (48.3%), had 1–3 children, while 102 (26.9%) had more than 3 children, and 94 (24.8%) had no children. A majority, 258 (68.1%), of respondents received the family planning method of their choice. Family planning methods used by the respondents included pills 72 (18.9%), injectable methods 151 (39.9%), implant methods 101 (26.7%), IUCD 50 (13.2%), and other methods 5 (1.3%).

Concerning method use explanation, 291 (76.8%) of respondents confirmed that the provider explained how to use the method, and 264 (69.7%) reported that potential side effects were explained for their chosen method. Additionally, 280 (73.9%) of the respondents reported that the provider offered a follow-up appointment if they encountered any problems with the method. Regarding waiting times, 221 (58.3%) of respondents waited less than 30 minutes for services, and a high proportion of respondents, 312 (82.6%), confirmed that their privacy was maintained during the procedure. Furthermore, 312 (82.3%) of respondents received their preferred method, and 292 (77%) experienced a respective provider during their visit. Finally, the distance from respondents' homes to the health facility was measured. A large portion, 172 (45.4%), of respondents lived within 30 minutes of the health facility, 159 (42%) lived 30–60 minutes away, and 48 (12.7%) lived more than 60 minutes away from the facility (Table 2).

**Table 2:** Clients' responses with aspects of family planning services and service provider-related factors (*n* = 379).

Variable	Category	Frequency	Percent (%)
Type of health facility	DTCSH	139	36.7
	Debre Tabor Health Center	99	26.3
	Leul Alemayehu Health Center	78	20.6
	Atse Seife Areid Health Center	63	16.4
Convenient service hours	Yes	293	77.3
	No	86	22.7
Frequency of visits	New	98	25.9
	Repeat	281	74.1
Number of children	No child	94	24.8
	1–3 Children	183	48.3
	More than 3 Children	102	26.9
Preferred method received	Yes	258	68.1
	No	121	31.9
Type of family planning method used	Pills	72	18.9
	Injectable	151	39.9
	Implant	101	26.7
	IUD	50	13.2
	Other	5	1.3
The method used is explained	Yes	291	76.8
	No	88	23.2
Side effects explained	Yes	264	69.7
	No	115	30.3
Follow-up appointment given	Yes	280	73.9
	No	99	26.1
Waiting time	<30 min	221	58.3
	30–60 min	122	32.2
	≥60 min	36	9.5
Privacy maintained	Yes	313	82.6
	No	66	17.4
Room cleanliness & informativeness	Yes	312	82.3
	No	67	17.7
Respectful provider	Yes	292	77
	No	87	23
Time taken from home to the facility	<30 min	172	45.4
	30–60 min	159	42
	≥ 60 min	48	12.7

### Family planning services satisfaction

**Figure 2:**
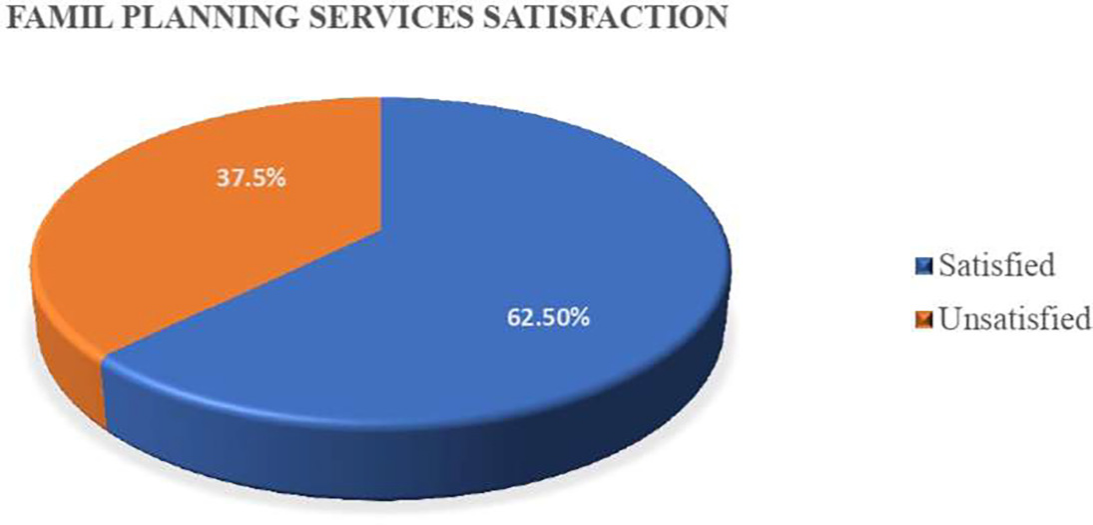
Satisfaction with family planning services among study participants.

Among the 379 respondents, 62.5% (95% CI: 58–67.5%) were satisfied with the family planning services provided (Figure 2).

### Factors associated with family planning services satisfaction

In the bivariate analysis, variables significantly associated with client satisfaction included educational status, marital status, occupation, residence, waiting time, method of counseling, follow-up appointment given, privacy maintained, preferred method received, and respectful provider.

The result from multivariable logistic regression indicated that government employees were 60% [AOR = 0.40; 95% CI: 0.18, 0.87; *p* = 0.020] less likely to be satisfied with family planning services compared to other occupations. Related to residence, clients residing in urban areas were about 1.68 times [AOR = 1.68; 95% CI: 1.08, 2.62; *p* = 0.022] more likely to be satisfied with the family planning service compared to rural residents. Clients who waited less than 30 minutes for services were 2.93 times [AOR = 2.93; 95% CI: 1.06, 8.07; *p* = 0.039] more likely to be satisfied than those who waited for more than an hour. Clients who received comprehensive method counseling were 2.32 times [AOR = 2.32; 95% CI: 1.29, 4.07; *p* = 0.005] more likely to be satisfied with family planning services compared with those who had not been informed about methods and side effects. Clients receiving the preferred method were 2.59 times [AOR = 2.59; 95% CI: 1.13, 3.94; *p* = 0.024] more likely to be satisfied with family planning compared with those who have not received the methods that the clients preferred to use (Table 3).

**Table 3:** Bivariate and multivariate analysis of factors associated with family planning services satisfaction (*n* = 379).

Variable	Category	Satisfaction		COR (95% CI)	AOR (95% CI)	*p*-value
		Satisfied	Dissatisfied			
Educational status	Informal education	76	54	1.00	1.00	-
	Formal education	161	88	0.78 (0.51–1.21)	1.28 (0.89–1.83)	0.812
Marital status	Married	193	91	0.80 (0.49–1.32)	1.42 (0.89–2.26)	0.140
	Unmarried	44	51	1.00	1.00	-
Occupation	Housewife	91	44	1.14 (0.68, 1.92)	1.04 (0.57, 1.89)	0.894
	Government employee	42	18	1.65 (1.01, 2.69)	0.40 (0.18, 0.87)	0.020*
	Private employee	35	19	1.08 (0.60, 1.95)	0.73 (0.32, 1.69)	0.460
	Merchant	35	33	0.95 (0.56, 1.60)	0.88 (0.41, 1.89)	0.745
	Others	34	28	1.00	1.00	-
Residence	Urban	212	117	1.84 (1.12, 3.02)	1.68 (1.08, 2.62)	0.022*
	Rural	25	25	1.00	1.00	-
Waiting time	<30 min	167	54	3.11 (1.78, 5.43)	2.93 (1.06, 8.07)	0.039*
	30–60 min	59	63	1.43 (0.74, 2.76)	1.21 (0.49, 2.99)	0.687
	≥60 min	11	25	1.00	1.00	-
Method counseling	Yes	190	60	4.98 (3.15, 7.88)	2.32 (1.29, 4.07)	0.005*
	No	47	82	1.00	1.00	-
Follow-up appointment given	Yes	209	71	5.45 (4.46, 7.44)	0.98 (0.52, 1.86)	0.954
	No	28	71	1.00	1.00	-
Privacy maintained	Yes	227	86	4.85 (3.33, 9.08)	1.52 (0.74, 3.12)	0.252
	No	10	56	1.00	1.00	-
Preferred method received	Yes	229	83	2.21 (1.24, 5.16)	2.59 (1.13, 3.94)	0.024*
	No	8	59	1.00	1.00	-
Respectful provider	Yes	217	75	0.15 (0.08, 0.28)	0.53 (0.28, 1.02)	0.071
	No	20	67	1.00	1.00	-

*p < 0.05, statistically significant.

### Sociodemographic and service provider-related characteristics of qualitative study participants

The majority of participants were aged 30–34 years, 5 (33.3%), and identified as Orthodox, 10 (66.7%). Most had completed primary school 4 (26.7%) and were married 10 (66.7%). Regarding occupation, the largest group was housewives, 5 (33.3%), with the majority residing in urban areas, 12 (80%). In terms of healthcare access, 6 (40%) participants visited DTCSH, 12 (80%) of participants reported convenient service hours, and 11 (73.3%) were repeat visitors. Most of the participants had 1–3 children 8 (53.3%), and 12 (80%) received their preferred family planning method (Table 4).

**Table 4:** Sociodemographic and service provider-related characteristics of qualitative study participants (*n* = 15).

Variable	Category	Frequency (%)
Age in years	15–19	3 (20%)
	20–24	4 (26.7%)
	30–34	5 (33.3%)
	>=35	3 (20%)
Religion	Orthodox	10 (66.7%)
	Muslim	3 (20%)
	Protestant	2 (13.3%)
Educational status	Cannot write/read	2 (13.3%)
	Read/write	3 (20%)
	Primary school	4 (26.7%)
	Secondary school	3 (20%)
	College & above	3 (20%)
Marital status	Single	2 (13.3%)
	Married	10 (66.7%)
	Divorced	2 (13.3%)
	Widowed	1 (6.7%)
Occupation	Housewife	5 (33.3%)
	Government employee	3 (20%)
	Private employee	3 (20%)
	Merchant	2 (13.3%)
	Other	2 (13.3%)
Residence	Urban	12 (80%)
	Rural	3 (20%)
Type of health facility	DTCSH	6 (40%)
	Health Center 1	3 (20%)
	Health Center 2	3 (20%)
	Health Center 3	3 (20%)
Convenient service hours	Yes	12 (80%)
	No	3 (20%)
Frequency of visits	New	4 (26.7%)
	Repeat	11 (73.3%)
Number of children	No child	3 (20%)
	1–3 children	8 (53.3%)
	3+ children	4 (26.7%)
Preferred method received	Yes	12 (80%)
	No	3 (20%)

### Client perception of family planning service

**Figure 3:**
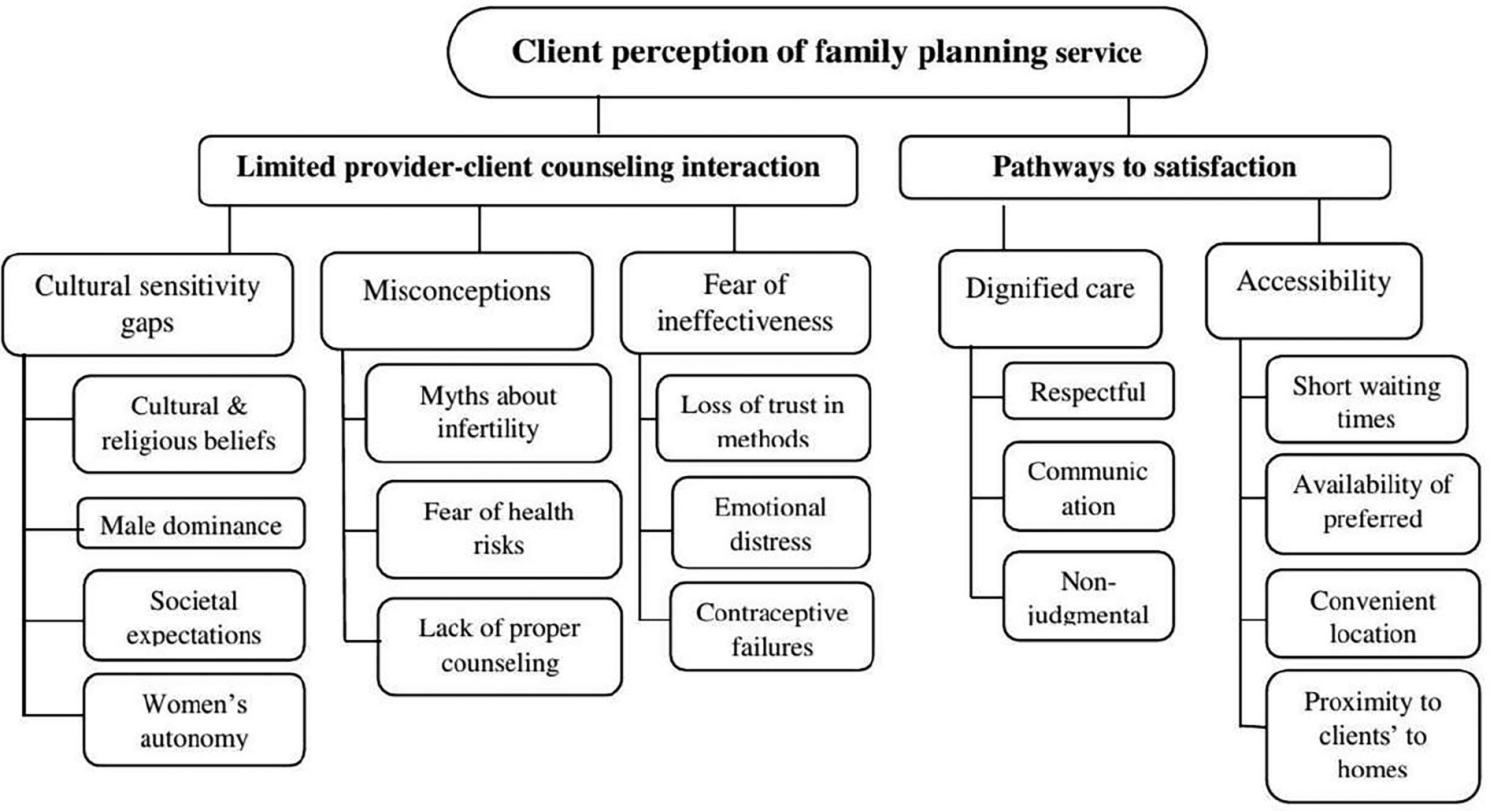
Coding tree for thematic analysis of clients' perceptions of family planning services.

Two themes were identified from the participants' in-depth interviews. Theme 1 reviews “limited provider-client counseling interaction,” which was subcategorized into “cultural sensitivity gaps,” “misconceptions,” and “fear of ineffectiveness.” Theme 2 addresses “Pathways to satisfaction,” which was subcategorized into “dignified care” and “accessibility” (Figure 3).

### Theme 1: limited provider-client counseling interaction

This theme addresses a critical gap in family planning counseling. Especially cultural, social, and religious perspectives that often lead clients to feel unsupported and dissatisfied; this affects the women's acceptance or continued use of modern methods. Notably, providers insufficiently address cultural-religious beliefs of society that affect clients' perceptions and decisions regarding family planning.

As Participant 6 shared, “*In our culture, women who stay home and have many children are respected. Yet, if a woman applies family planning or has a few children, society calls her lazy. I chose to use family planning, but I lost the respect of my community. I needed someone to talk to about these cultural pressures and to feel sure I was making the right choice.*” This quote underlines the cultural and familial pressures women face, where the expectation to have many children is supported by societal beliefs. Also, it further reflects how family planning is stigmatized and lacks the encouragement or guidance needed to feel confident in using these methods. This theme was subcategorized into three subthemes: “cultural sensitivity gaps,” “misconceptions,” and “fear of ineffectiveness.”

#### Subtheme 1.1: cultural sensitivity gaps

Family planning counseling often fails to address cultural values and beliefs that influence clients' perception of reproductive health, leading to client dissatisfaction. Traditional beliefs about family size, gender roles, and male authority create barriers, making clients feel misunderstood, unsupported, and alienated during interactions with providers.

A key sensitivity gap is the role of gender dynamics, particularly male dominance in decision-making, which often limits women's autonomy in accessing family planning services. Men are often the primary decision-makers, and women often require consent from their husbands. This cultural sensitivity gap is renewed by misconceptions about contraceptives, stigma, and household conflict. This cultural pressure and male dominance highlighted by Participant 3, “*I wanted to use family planning, but my husband was opposed, saying it was unnatural and that we should have as many children as possible, like his mother, who had 8 children, and his grandmother, who had 12. I didn't feel empowered to decide, but eventually, I managed to persuade him to use the implant to avoid a fifth pregnancy.*” This statement revealed the gender dynamics in family planning decisions. In many communities, men hold the dominant role in reproductive decisions, and their resistance to family planning can limit women's autonomy. Similarly, Participant 9 echoed, “*I hide my visits to the clinic from my husband since he believes one becomes rich through a multitude of children. But I believe that I must work and become an independent woman to give my children the necessary materials, education, and a better future. Presently, I am just a housewife*.” This shows the tension between tradition and the expectations placed upon women, compared with their desire for independence and improved prospects for their children. Another participant, 5, explained, “*When I told the nurse that I was afraid my husband would not agree to family planning, she just told me, ‘Talk to him,’ but she did not help me think of a way*.” This shows how a lack of culturally sensitive counseling leaves women feeling misunderstood, disempowered, and less likely to engage with reproductive health services.

#### Subtheme 1.2: misconceptions

Misconceptions about family planning methods include the perception that they cause infertility, serious illnesses like cancer, marital conflict, reduced sexual desire, and impaired future pregnancies. These concerns are rarely addressed during counseling sessions, leaving a critical gap that allows misinformation to persist and creates frustration and a lack of confidence in the services, ultimately leading to dissatisfaction and reduced utilization.

Participant 1 shared, “*In our community, people argued that family planning is against God's plan, believing that God should decide when to stop having children. If continued, God may punish those who continue using it with infertility.”* This reflects how familial influence and misconceptions affect seeking family planning.

Another participant, 13, who married three months ago, expressed concerns about the myths she had heard regarding injectable contraception, particularly the fear that it could lead to infertility: “*I have heard that using family planning methods when you haven't had children yet can cause infertility. I'm really afraid to take contraceptives because of that.*” This reflects common misconceptions about infertility and the use of contraceptives, especially among those who have not had children. The participant's fear is fueled by societal myths and a lack of proper counseling, which further discourages and dissatisfies the use of family planning methods.

Additionally, participant 4 described an unexpected side effect: “*No one advised me that family planning could cause weight gain, especially around my belly. I didn't know about this side effect, and now I feel uncomfortable and doubt the validity of community claims about its potential harm to women's health*.” The participant's frustration underscores the communication gap between providers and participants regarding side effects of family planning methods, highlighting the need for clear, empathetic communication to address myths and empower clients.

#### Subtheme 1.3: fear of ineffectiveness

A gap in family planning counseling is the failure to give sufficient attention to clients' fears about the effectiveness of the contraceptive methods. This often results in feelings of betrayal, reduced trust in contraception, and emotional distress that leaves the clients frustrated, confused, and unwilling either to continue with the same method or to try other methods. Participant 10 narrated, “*My fourth pregnancy was unplanned, and it happened while I was using a method. I named him Behailu, which means ‘his power.’ I felt like I was forced into this situation, even though I thought I was being careful.*” This powerful statement captures the emotional toll that contraceptive failure can take.

Another participant, 12, described, “*I used the method, but I ended up pregnant anyway. To my surprise, I didn't know I was pregnant until the fourth month because I trusted the method. When I started feeling strange in my abdomen, I thought it was an illness, so I took holy water for three weeks. Later, when I went to a clinic, I was shocked to hear I was pregnant. I couldn't believe it and was scared for my baby's health. Now, I'm really scared of trying anything else; I just don't have any trust anymore in contraception, though I take the method since it is the only option I am left with*.” This quote further illustrates the emotional turmoil clients experience after a contraceptive method fails, emphasizing the need for comprehensive family planning counseling that addresses both theoretical and emotional aspects, fostering trust and informed decision-making.

### Theme 2: pathways to satisfaction

Empowerment in family planning services is about creating an environment where clients feel supported, informed, and confident in their decisions. Providing personalized care to meet emotional and practical needs can help healthcare providers create closer relationships with clients. Respectful communication, understanding of client preferences, and accessible and efficient service will help clients feel more in control of their reproductive health choices. When tools, knowledge, and support are provided for clients, they are in a position to feel more empowered, thus increasing satisfaction and adherence to a family planning method, which will increase the likelihood of return for future care. Participant 2 shared, “*I have received family planning service from this health center since I got married. I have two kids within 8 years, and I am so happy with the service. The clinic is just a few meters from my home. The staff are friendly, and they ensure that I understand well*.” It therefore demonstrated that easy access, respect, added clarity in communication, and convenient services are some of the key reasons these clients have confidence in their decisions and hence are more likely to continue family planning. This theme is further divided into two subthemes, such as “dignified care” and “accessibility.”

#### Subtheme 2.1: dignified care

This subtheme highlights that respectful interaction plays a vital role in gaining confidence and building a supportive environment, which is very important for the satisfaction of clients in sensitive areas of service delivery like family planning. When healthcare providers approach their clients in a respectful, non-judgmental, and caring way, clients feel listened to and valued. Such interactions will add not only to the current experience of care but also build trust that will last, enabling clients to come back for other visits and inform others about the service. Listening on the part of the health care provider and taking time to explain the details of care reassures and empowers clients in making fully informed decisions about their reproductive health.

Participant 11 shared, “*I am here today for a consultation after getting the IUD. It is causing some confusion and discomfort, but the professionals are good at explaining everything. They also told me to have it checked again in case there was some other problem*.” This, therefore, shows the ability of clear communication and assurance by healthcare providers in handling apprehension and ensuring that clients do not feel uneasy coming back for further care. Another participant, 8, commented, “*The provider was patient, explained everything clearly, and never rushed or judged me; I felt at complete ease*.” This emphasizes that clear and kind words are all a client requires to create trust and a good relationship with the provider.

#### Subtheme 2.2: accessibility

Clients' satisfaction with family planning services is greatly influenced by the accessibility of these services, which includes factors like convenient clinic hours, short waiting times, and the availability of preferred contraceptive methods. For the efficiency of health services to be realized and made easily available, clients will have more confidence in incorporating visits for family planning into their routine and overcoming most of the barriers to care. When clients can receive their method of choice, not having to choose an alternative option, this empowers a person's self-efficacy in reproductive decision-making, hence leading to higher satisfaction. Participant 7 reported, “*I appreciate how quickly I was seen. It didn't take long at all, so I'm more likely to return*.” This again points to the relevance of timely service to foster continued use.

As Participant 14 said, “*I arrived in this town a year ago, and the clinic opens early, so I can go before work. It is a big help, especially with my busy schedule*.” This emphasizes how flexible clinic hours are important for those with busy schedules and thus make access to the services easier. This is the reason why empathetic care, coupled with accessible and efficient services, not only enhances the trust of the clients but also empowers them toward confident, fully informed decisions about family planning, with long-term adherence to the respective services.

### Integrated quantitative and qualitative findings on client satisfaction with family planning services

The integration of quantitative and qualitative data revealed several convergent findings, which mutually enhanced the understanding of family planning satisfaction. Quantitative analysis showed that 62.5% of clients were satisfied with the services, and this satisfaction was significantly associated with urban residence, shorter waiting times, and preferred method received. These findings were echoed in the qualitative sub-themes of accessibility and dignified care, in which respectful communication, client empowerment, and autonomy were emphasized. Notably, a divergence emerged: the quantitative findings showed a positive association between method counseling and client satisfaction, whereas the qualitative insights identified dissatisfaction with counseling—marked by insufficient detail, unresolved misconceptions, and unmet informational needs.

In addition, each method yielded unique findings. Quantitative data revealed a significant dissatisfaction among government employees, yet these concerns were not specifically captured in qualitative narratives. Conversely, qualitative findings identified issues not measured quantitatively, including cultural sensitivity gaps, misconceptions, and fear of ineffectiveness of contraceptives (Table 5).

**Table 5:** Joint display of integrated quantitative and qualitative findings on family planning service satisfaction.

Quantitative findings	Qualitative Theme/Sub-themes	Integration Type	Integrated interpretation
62.5% satisfied overall	Theme: Pathways to satisfaction	Convergence	Satisfaction was reflected in narratives of respectful communication, accessible services, and method choice, supporting the quantitative satisfaction rate.
	*Sub-themes: Dignified care & accessibility*		
Government employees' ↓satisfaction [AOR = 0.40]	—	Quantitative unique finding	Government employees had significantly lower satisfaction with family planning services compared to other occupations. This subgroup-specific dissatisfaction was not identified in qualitative data, possibly due to government employees being educated and having higher expectations for unmet needs, resulting in lower quantitative satisfaction.
Urban residents' ↑ satisfaction [AOR = 1.68]	Theme: Pathways to satisfaction	Convergence	Physical access, shorter travel time, and flexible clinic hours are consistent with quantitative results showing higher satisfaction in urban areas.
	*Sub-theme: Accessibility*		
Waiting time <30 min ↑ satisfaction [AOR = 2.93]	Theme: Pathways to satisfaction	Convergence	Both data sets confirm that shorter waiting times are crucial for satisfaction. Participants emphasize that quick, efficient service encourages continued use.
	*Sub-theme: Accessibility*		
Method counseling ↑ satisfaction [AOR = 2.32]	Theme: Limited provider-client counseling interaction	Divergence	Quantitative data shows counseling increases satisfaction; qualitative data explains dissatisfaction arises when counseling is limited, leaving myths and fears unaddressed. Together, they highlight counseling quality as key to satisfaction.
	*Sub-themes: Misconceptions, Fear of ineffectiveness*		
Receiving the preferred method ↑ satisfaction [AOR = 2.59]	Theme: Pathways to satisfaction	Convergence	Receiving the preferred contraceptive method empowers clients, builds trust, and increases satisfaction, confirmed by both quantitative association and client narratives.
	*Sub-theme: Dignified care*		
—	Theme: Limited provider-client counseling interaction *Sub-themes: Cultural sensitivity gaps, misconceptions, and fear of ineffectiveness*	Qualitative unique finding	Gender dynamics, male authority, myths about contraception, and emotional distress in unplanned pregnancies are largely qualitative, highlighting sociocultural barriers to women's autonomy and highlighting a counseling gap.

↑ = increased or positive association; ↓ = decreased or negative association.

## Discussion

Clients' satisfaction is the key indicator for healthcare quality, particularly within the domain of reproductive health services (7). In this convergent mixed-methods study, the quantitative finding revealed that 62.5% (95% CI: 58–67.5%) of respondents were satisfied with family planning services provided by public health facilities. This result aligns with findings from Bahir Dar city, Northwest Ethiopia (66.1%) (19), Pawi district, Ethiopia (60.5%) (30), and Kenya (61%) (31), suggesting comparable levels of service quality and accessibility in similar settings. However, the satisfaction rate in this study is higher than those reported in Tembaro District, Southern Ethiopia (46%) (9), Jigjiga town, Eastern Ethiopia (41.7%) (21), and Pakistan (33%) (32). This may be related to better health infrastructure, differences in study design, and measurement tools. These were strongly reinforced by the qualitative theme “pathways to satisfaction,” which explores the accessibility, respectful care, and participants' active participation. This insight also aligns with a qualitative study in Kenya (33), where respectful, welcoming provider interactions and privacy/confidentiality are key dimensions of perceived family planning services quality.

Conversely, the satisfaction rate in this study is lower than those reported in several Ethiopian towns, such as Kuncha District (68.4%) (14), Halaba Town (71.2%) (34), and Hosanna Town (75.3%) (7), as well as in other countries including Nigeria (85%) (20), Mozambique (86%) (35), and Port Harcourt, Nigeria (87.2%) (36). These higher levels of satisfaction may be related to facility readiness, provider competency, and client-centered approaches. This was further explored by the qualitative data theme “limited provider-client interaction,” arguing that while the providers technically inform, they fail to address cultural sensitivity and contextually understood issues, leaving participants dissatisfied. Another qualitative study in Burkina Faso stated that failure to address cultural norms, misconceptions, and stigma reduced perceived service quality and satisfaction (37).

One of the significant findings of this study was that urban residents were more likely to be satisfied with the family planning service than rural residents. This finding is in agreement with studies in Ethiopia (38) and Kenya (39). The possible reason is that urban residents often benefit from better service availability, socio-economic opportunities, shorter travel distances, timely services, and convenient clinic hours, which can positively influence their satisfaction (40). This finding is also supported by the qualitative data sub-theme of “Accessibility,” which stated that flexible service hours and shorter waiting times are repeatedly linked to greater trust and satisfaction. This result aligns with qualitative research from Uganda's Kira municipality (41), where women living in informal urban settlements reported lower satisfaction with family planning services compared to better-resourced formal settlements, while proximity to well-staffed clinics and shorter wait times increases trust and satisfaction.

Another notable finding of this study is that clients employed in government sectors reported a lower level of satisfaction with family planning services than those in other occupations. This result is supported by studies conducted in Bahir Dar City, Northwest Ethiopia (19), the West Shoa Zone, Central Ethiopia (42), and Mozambique (43). The possible reason might be that the governmental working hours and family planning service hours were the same, and the overlap of these might lead to government employees being dissatisfied with service hours. Additionally, government employees are educated, so they may hold higher expectations for personalized, comprehensive, and culturally sensitive counseling. When these expectations are unmet, it can lead to a heightened sense of dissatisfaction, amplified by work-related stress and time constraints.

This study revealed that clients who waited for less than 30 minutes were more likely to be satisfied with family planning services than those who waited over an hour. This finding is consistent with studies conducted in various parts of Ethiopia, including Bahir Dar city (19), Tembaro District (9), Hosanna town (7), and Halaba town (35), as well as in Sokoto, Nigeria (20), and Mozambique (35). These consistent results suggested that shortening waiting times could increase client satisfaction and improve the quality of family planning services. The qualitative findings further stated that timely service fosters continued use, and flexible clinic hours enable clients with busy schedules to access care more easily. These findings are also supported by a study in Western Kenya (44), which utilized focus groups, key informant interviews, and mystery client visits, stating that long waiting times and rushed consultations were key sources of dissatisfaction.

In the current study, methods counseling was significantly associated with satisfaction with family planning services. This finding is consistent with studies conducted in Tembaro District (9) and Jigjiga town (21), Ethiopia. The possible explanation is that counseling plays a key role in ensuring the quality of family planning service delivery by facilitating informed choice, addressing fears or misconceptions, and building trust between clients and providers. The Ethiopian national family planning guidelines emphasize the importance of comprehensive counseling for enhancing service utilization and satisfaction and reducing discontinuation rates. The above findings are also reinforced in the qualitative study under the theme of “Pathways to satisfaction”, Clients feel empowered when providers listen, explain options without judgment, and address their concerns. Effective counseling is a relational process essential for quality care and long-term engagement in family planning. Another qualitative study from Southeast Ethiopia also verified counseling linked to higher satisfaction and trust in family planning services (16).

The present study also revealed that clients who received their preferred family planning method were more likely to be satisfied with family planning services. This study was in agreement with previous studies conducted in Jigjiga town, Eastern Ethiopia (21) and Mozambique (35). The possible reason is that receiving a preferred method enhances a sense of autonomy and control, which is employed to gain greater satisfaction. The result is further asserted through the qualitative findings: when participants' preferences were respected and they were actively involved in the decision, they were expressed more trust in the service and great satisfaction. This is echoed by qualitative research conducted in Adigrat Town, Northern Ethiopia (45), and Kenya (46), where receiving a preferred method empowered clients, making them confident and satisfied with the services.

Furthermore, the qualitative study explored a critical gap in family planning services, particularly the inadequate attention given to cultural, social, and religious perceptions. In the Ethiopian context, deeply rooted social norms—such as male dominance in decision-making, religious interpretations surrounding fertility, and societal stigma against contraception—continue to hinder open discussions about reproductive choices. Despite national health policies such as the Health Sector Transformation Plan (HSTP) emphasizing equity and culturally sensitive service delivery, implementation remains inconsistent, especially at the primary healthcare level. Women often feel unsupported when providers fail to address these cultural sensitivities, misconceptions, and fears about contraceptive effectiveness. This lack of culturally sensitive counseling leads to dissatisfaction, ultimately jeopardizing contraceptive acceptance and continuation. Similar themes have emerged in other qualitative studies conducted in Uganda (47) and Jordan (48) showing that cultural beliefs and gender dynamics strongly influence contraceptive use, and addressing misconceptions and providing empathetic, culturally tailored counseling improves client trust and satisfaction.

## Conclusion and recommendations

This mixed-methods study revealed that client satisfaction with family planning services in public health facilities remains moderate, with significant disparities in residence, occupation, waiting time, method counselling, and receiving a preferred method. Also, the qualitative study emerges two main themes: limited provider-client counseling interaction and pathways to satisfaction. Clients reported that counseling often lacked cultural sensitivity, failed to address misconceptions, and did not involve them in decision-making—undermining trust and satisfaction. On the other hand, satisfaction was higher when services were respectful, timely, and responsive to clients' preferences. Accessible services and dignified care helped foster trust and supported continued family planning use.

To enhance family planning service satisfaction, health facilities should strengthen client-centered counseling by addressing cultural misconceptions, ensuring respectful communication, and actively involving clients in method selection. Expanding flexible service hours and reducing waiting times are also vital to meet the needs of working clients. Continuous training for providers on effective, culturally sensitive counseling techniques is essential to build trust and improve overall service quality.

## Strengths and limitations

The study employed a mixed-methods approach, which allowed a comprehensive assessment of client satisfaction. Quantitative data provides measurable information about satisfaction determinants, while qualitative findings provide detailed, nuanced explanations that maintain the client voice and experience in greater depth.

The study has limitations. First, its cross-sectional design limits the ability to establish causal relationships between the associated factors and client satisfaction. Second, the study was conducted exclusively in public health facilities, potentially excluding dissatisfied clients who may have avoided these settings. This selection bias could result in an overestimation of the overall satisfaction levels. There is also a possibility of social desirability bias, as participants may have under-/over-reported satisfaction during face-to-face interviews particularly in response to Likert scale items.

## Data Availability

The raw data supporting the conclusions of this article will be made available by the authors, without undue reservation.
