# Supplementary material for: Exploring client satisfaction and determinants of family planning services at public health facilities in Debre Tabor town, Northwest Ethiopia: a mixed-method study
Source: Front Reprod Health. 2025 Aug 29;7:1558606. doi: 10.3389/frph.2025.1558606 (PMC12426020; doi:10.3389/frph.2025.1558606)
Supplement: Supplementary file 1 [file Datasheet1.pdf]

Exploring client satisfaction and determinants of family planning services at public health facilities in Debre Tabor Town, Northwest Ethiopia, 2024

Introduction

Globally, 0.35 billion couples are unable to access or are limited to modern family planning and this situation is severe in sub-Saharan African countries. An estimated 222 million women in low- and middle-income countries have an unmet need for modern contraception. Around 150 million married women in the developing world want to delay or stop childbearing, but they do not have access to modern contraception [1-3].

In 2012, there were estimated to be more than 74 million unintended pregnancies in the developing world, including 4.6 million in West Africa. Sub-Saharan Africa (SSA) has the highest fertility rate in the world and the highest unmet need for family planning (FP). In Ethiopia, FP was initiated four decades ago; however, even after such a long period of time, the service has been among the lowest in Africa, with 36% unmet need for FP, while the already available services do not appear to be optimally used by potential clients [4-7].

According to the Ethiopian Demographic and Health Survey (EDHS) 2016 report, 22% of currently married women have an unmet need for family planning. In addition, more than one-third (35%) of women who commence contraception discontinue use within 12 months. The Ethiopian government introduced the Health Sector Transformation Plan (HSTP) intending to increase the contraceptive prevalence rate (CPR) to 55% and reduce the unmet need for FP use to 10% by 2020, but the progress is still slow. The unmet need for family planning among

reproductive-aged women in rural Ethiopia was high. The overall unmet need for family planning among those was 24.08% [8-10].

Client satisfaction was increased by specific interventions, namely, shortening the waiting time, maintaining privacy during counselling and procedures, equipping service provision rooms with necessary materials, improving counselling specifically on the side effects of the family planning methods, having a family planning room with posters displaying key messages on family planning, having a discussion with husbands about the family planning methods, describing how the methods work, and making an appointment for the next visit to family planning [10, 11].

Client satisfaction is considered one of the desired outcomes of health care and is directly related to the utilization of health services. However, different factors are found to affect client satisfaction, such as waiting time, kind of provider, privacy, staff motivation, poor quality of care, availability of medicines and equipment, and some demographic characteristics like the age and education of the client [9, 11, 12].

2. Objectives

2.1. General Objective

To assess client satisfaction and explore determinants of family planning services at public health facilities in Debre Tabor Town

2.2. Specific Objectives

To measure the level of client satisfaction with family planning services.

To identify socio-demographic, organizational, and service-related factors influencing client satisfaction.

To explore the lived experiences of clients regarding family planning services through qualitative inquiry.

3. Methods and materials

3.1 Study design

A mixed-method study design, incorporating both facility-based cross-sectional and phenomenological study approaches will be conducted

3.2 Study period

The data collection will take place from April to May, 2024.

3.3 Study setting

The study will be conducted in public health facilities of Debre Tabor Town, Northwest Ethiopia. Debre Tabor town administration is one of the 13 districts and five town administrations found in the South Gondar administrative zone in Ethiopia. It is located 105 km away from Bahir Dar and 666 km away from Addis Ababa. The town has a total population of 83,082, of whom 39,781 are males and 43,301 are females (BOFED 2015). The total number of households in the town is 13,200, and the town has one referral hospital, known as Debre Tabor Comprehensive Specialized Hospital (DTCSH), and three health centres, namely Debre Tabor Health Center, Leul Alemayehu Health Center, and Atse Seife Areid Health Center. These public health institutions are currently providing various health services, including family planning services, for the residents of Debre Tabor and people around the town.

3.4 Study population

3.4.1. Source population

All reproductive age women attending family planning services at selected health facilities

3.4.2. Study population

All reproductive-age women attending family planning services at selected health facilities during data collection period

3.5 Inclusion and exclusion criteria

Women aged 15–49 who received family planning services will be included in the study. While female health care's workers who take a family planning services in the facilities will be excluded.

3.6 Sampling

3.6.1. Sample Size Determination

The sample size for the quantitative part will be calculated using the single population proportion formula, considering:

The sample size is determined by using single population proportion formula by considering the following statistical assumptions: 95% confidence interval (CI), 66.1%proportion (from previous Ethiopian study at Bahir Dar city, Public Health facilities,2017 [12])

$$ni = \frac{(z)^2 P(1-P)}{d^2}$$

Where, ni= initial sample size

Z= 1.96 the corresponding Z-score for the 95% CI

P= Proportion 66.1% (from previous Ethiopian study, 2017) = 0.661

d= Margin of error= 5%= 0.05

$$\underline{ni} = \frac{(1.96)2 \text{ x } 0.661(1-0.661)}{(0.05)2} = 344$$

An additional 10% will be added to account for non-response, resulting in 379 participants.

4.2 Sampling Procedure

A multi-stage stratified random sampling technique will be employed. For the qualitative part, participants will be selected using purposive sampling until data saturation is achieved.

Dependent Variable

Client satisfaction level (satisfied/dissatisfied)

Independent Variables

Socio demographic variables: - Age, education, resident, marital status, religion, ethnicity, occupation, income

Obestetrics and health service related variables:- Waiting time, unintended pregnancy, side effects, information given, privacy, cleanlines of facility and room and avalibilty of contraceptive metheds

5. Data Collection

Data for the study will be collected using a combination of quantitative and qualitative approaches to ensure a comprehensive understanding of client satisfaction and its determinants. A structured interviewer-administered questionnaire adapted from different literatures [1, 12-18]. Interviewer-administered questionnaires will be used to gather information on socio-demographic characteristics, organizational factors, and client satisfaction levels. These questionnaires will be pre-tested on 5% of the sample in a nearby health facility not included in the study to ensure clarity, reliability, and validity. Trained data collectors with prior experience will administer the questionnaires, ensuring participants' anonymity and confidentiality by assigning unique codes and avoiding the collection of personally identifiable information. Supervisors will review completed questionnaires daily to check for completeness and consistency, addressing any gaps promptly.

For the qualitative component, semi-structured interview guides will be used to conduct in-depth interviews with purposefully selected participants. These interviews will explore lived experiences with family planning services, including cultural influences, counseling gaps, and perceptions of service quality. To ensure privacy and encourage openness, interviews will be

conducted in private rooms within the health facilities. Each interview will be audio-recorded with participants' consent and supplemented by detailed field notes. Transcriptions of the recordings will be prepared verbatim and translated into English for analysis.

To maintain high data quality, data collection teams will undergo intensive two-day training on the study objectives, ethical considerations, and administration of data collection tools. Supervisors will provide continuous oversight and feedback during the data collection period to ensure accuracy and consistency.

6. Data Analysis

The data that obtained from each participant in quantitative study will be checked for its completeness and clearness. And then data entry will be performed by using computer software namely Epi info and will be analyzed by SPSS. Descriptive statistics will be used to represent participant characteristics through the use of tables, graphs, and figures. Bivariable and multivariable analyses will be performed. Thematic analysis will also be used for qualitative data through transcription, coding, and theme generation.

8. Dissemination of Results

The findings will be disseminated through presentations, publications, and reports shared with relevant stakeholders and the health facilities involved.

References

1. Alie, M.S., G.F. Abebe, and Y. Negesse, *Magnitude and determinants of unmet need for family planning among reproductive age women in East Africa: multilevel analysis of recent demographic and health survey data.* Contraception and Reproductive Medicine, 2022. **7**(1): p. 1-11.
2. Machiyama, K., et al., *Reasons for unmet need for family planning, with attention to the measurement of fertility preferences: protocol for a multi-site cohort study.* Reproductive health, 2017. **14**(1): p. 1-11.
3. Wolde, A., et al., *Unmet need for modern contraceptive methods and associated factors among currently married women in Damot Woyde District, SNNPR, Ethiopia, 2019.* Open Access Journal of Contraception, 2020: p. 177-185.
4. Staveteig, S., *Fear, opposition, ambivalence, and omission: results from a follow-up study on unmet need for family planning in Ghana.* PloS one, 2017. **12**(7): p. e0182076.
5. Gahungu, J., M. Vahdaninia, and P.R. Regmi, *The unmet needs for modern family planning methods among postpartum women in Sub-Saharan Africa: a systematic review of the literature.* Reproductive Health, 2021. **18**: p. 1-15.
6. Kassa, M., A.A. Abajobir, and M. Gedefaw, *Level of male involvement and associated factors in family planning services utilization among married men in Debremarkos town, Northwest Ethiopia.* BMC international health and human rights, 2014. **14**: p. 1-8.
7. Argago, T.G., K.W. Hajito, and S.B. Kitila, *Clients satisfaction with family planning services and associated factors among family planning users in Hossana town public health facilities, South Ethiopia: facility-based cross-sectional study.* International journal of Nursing and Midwifery, 2015. **7**(5): p. 74-83.
8. Fantahun, M., *Quality of family planning services in northwest Ethiopia.* Ethiopian Journal of Health Development, 2005. **19**(3): p. 195-202.
9. Wogu, D., T. Lolaso, and M. Meskele, *Client satisfaction with family planning services and associated factors in Tembaro District, Southern Ethiopia.* Open Access Journal of Contraception, 2020: p. 69-76.
10. Alem, A.Z. and C.D. Agegnehu, *Magnitude and associated factors of unmet need for family planning among rural women in Ethiopia: a multilevel cross-sectional analysis.* BMJ open, 2021. **11**(4): p. e044060.
11. Bintabara, D., et al., *Client satisfaction with family planning services in the area of high unmet need: evidence from Tanzania Service Provision Assessment Survey, 2014-2015.* Reproductive health, 2018. **15**: p. 1-9.
12. Asrat, W., T. Mekonnen, and M. Bedimo, *Assessment of women's satisfaction with family planning service at public health facilities in Northwest Region of Ethiopia: a cross sectional study.* Contraception and reproductive medicine, 2018. **3**(1): p. 1-8. 13. Wogu, D., T. Lolaso, and M. Meskele, *Client satisfaction with family planning services and associated factors in Tembaro District, Southern Ethiopia.* Open Access Journal of Contraception, 2020. **11**: p. 69.
14. Kaoje, U.A., et al., *Determinants of client satisfaction with family planning services in government health facilities in Sokoto, Northern Nigeria.* Sahel Medical Journal, 2015. **18**(1): p. 20.

15. Gebreyesus, A., *Determinants of client satisfaction with family planning services in public health facilities of Jigjiga town, Eastern Ethiopia.* BMC health services research, 2019. **19**(1): p. 1-10.
16. Wai, M.M., et al., *Unmet need for family planning among urban and rural married women in Yangon region, Myanmar—a cross-sectional study.* International journal of environmental research and public health, 2019. **16**(19): p. 3742.
17. Tsegaye, G.A., W.H. Kifle, and B.K. Sena, *Clientâ€™ s satisfaction with family planning services and associated factors among family planning users in Hossana Town Public Health Facilities, South Ethiopia: Facility-based cross-sectional study.* International journal of Nursing and Midwifery, 2015. **7**(5): p. 74-83.
18. Hutchinson, P.L., M. Do, and S. Agha, *Measuring client satisfaction and the quality of family planning services: a comparative analysis of public and private health facilities in Tanzania, Kenya and Ghana.* BMC health services research, 2011. **11**(1): p. 1-17.
