# Supplementary material for: Exploring client satisfaction and determinants of family planning services at public health facilities in Debre Tabor town, Northwest Ethiopia: a mixed-method study
Source: Front Reprod Health. 2025 Aug 29;7:1558606. doi: 10.3389/frph.2025.1558606 (PMC12426020; doi:10.3389/frph.2025.1558606)
Supplement: Supplementary file 2 [file Datasheet2.pdf]

Annex 1: Research participant information sheet in English version

Introduction:

An invitation to participate in a research project has been extended to you. It's critical that you comprehend the purpose and scope of the research before deciding whether or not to participate. Please listen to the following information carefully for a few minutes. Ask me any questions you have or if you'd like further details.

Title:

Exploring client satisfaction and determinants of family planning services at public health facilities in Debre Tabor Town, Northwest Ethiopia

Purpose of the study:

The purpose of this study is to assess client satisfaction with family planning services at public health facilities and to explore the factors influencing satisfaction. Your participation will help us better understand how these services can be improved.

Why are you the one I chose?

As a client who is taking a service in this health facility, you were selected to participate in this study. The project has received ethical clearance after assessment by the Ethics Approval Committee and also the principal has approved the conduct of this study.

What participation involves

If you agree to participate in this study, you will be asked questions about your experiences with family planning services. The interview or survey will take approximately 20–30 minutes of your time. Your responses will be recorded anonymously, and no personal identifiers, such as your name or contact information, will be linked to the data collected.

Voluntary participation

Participation in this study is strictly on a volunteer basis. You are free to withdraw from this study at any time, and you do not need to give any reason for doing so. You may refuse to answer any question if it makes you uncomfortable. You are not subject to any penalties or loss of benefits to which you are otherwise entitled by refusing to participate in this study.

Risks and benefits

No risk to you participating in this research study is perceived. You yourself might not obtain direct benefits through the research procedure; however, the result from this research might play an immense role for family planning services improvements in general at Debre Tabor Town and beyond.

Confidentiality

Your privacy is guaranteed, and no information that would disclose your identity will be retained from this study. No recording of personally identifiable information will be kept during this study. The data will be safely kept and accessed only by the research team. Furthermore, the study results will be reported in a way that ensures no individual participant can be identified. In addition, the research will be published in academic journals.

Contact information

If you have any questions about the study or your rights as a participant, please contact:

Investigator(s): Tseganesh Asefa, Winta Tesfaye, Gedamnesh Bitew, Hiwot Tezera

Principal Investigator:

Tseganesh Asefa

Phone No: +251922736330, +251715050533

Email: tseganesh16@gmail.com / tseganesh199@gmail.com

Annex II: Research participant consent form

I have read (or had read to me) the information above. I have had the opportunity to ask questions, and my questions have been answered. I understand that my participation is voluntary and that I can withdraw at any time without penalty.

I agree to participate in this study.

Participant's signature or thumbprint: _______________________

Date: _______________

Name of interviewer (if applicable): _________________________
