# Supplementary material for: Exploring client satisfaction and determinants of family planning services at public health facilities in Debre Tabor town, Northwest Ethiopia: a mixed-method study
Source: Front Reprod Health. 2025 Aug 29;7:1558606. doi: 10.3389/frph.2025.1558606 (PMC12426020; doi:10.3389/frph.2025.1558606)
Supplement: Supplementary file 3 [file Datasheet3.pdf]

Annex III: Questionnaire

Introduction: - Dear clients, my name is ______________________, a data collector of this study. First I would like to thank you for your voluntary participating in this study. I kindly asked that you answer the interview truthfully, and I guarantee that both your response and any personally identifiable information will be kept private. Your insights will provide valuable information for improving support and interventions for individuals facing similar experiences.

Part one: Structured English version questioner about socio-demographic characteristics

**Part I: Socio demographic characteristics**		
No	Question	Coding category
1)	How old are you?	Age in years __________________
2)	What is your religion?	1. Orthodox 3. Protestant<br>2. Muslim 4. Catholic<br>5. Others specify…….
3)	Your educational level?	1. Cannot write and read<br>2. Only read and write<br>3. Primary (1-8) school<br>4. Secondary(9-12) school<br>5. College/university and above
4)	Your marital status?	1. Single 3. Divorced<br>2. Married 4. Widowed
5)	Your occupation?	1. Housewife 5. Daily laborer<br>2. Gov't employee 6. Student<br>3. Private employee 7. Merchant<br>4. Jobless 8. Others specify_______
6)	Your annual income	__________________ETB
7)	Your residence?	1) Urban<br>2) Rural

Part III:- Organization and service provider-related questions		
1.	Which health facility do you use for family planning services?	1. Debre Tabor Comprehensive Specialized Hospital (DTCSH)<br>2. Debre Tabor Health Center<br>3. Leul Alemayehu Health Center<br>4. Atse Seife Areid Health Center
2.	Are the opening hours of this facility convenient for you?	1. Yes 2. No
3.	How often do you visit?	1. New 2. Repeat
4.	How many numbers of children do you have?	____________
5.	Have you received the method you want?	1. Yes 2. No
6.	Which type of FP method did you use?	1. Pills 3. Implant<br>2. Injectable 4. IUD<br>5. Other
7.	Were you told how to use the method you received?	1. Yes 2. No
8.	Were you told about the methods' side effects?	1. Yes 2. No
9.	Did the provider tell you to return if you have a problem?	1. Yes 2. No
10.	How much you wait between you first arrived here and the service you get?	1. <30 minutes<br>2. 30minutes- 1hour<br>3. $\geq$1hour
11.	Was there enough privacy during consultation and examination?	1. Yes 2. No
12.	Is the room clean and informative about the methods?	1. Yes 2. No
13.	Were you welcomed by the provider when you arrived here first?	1. Yes 2. No
14.	How long does it take to travel from your home to the health facility?	1. <30 minutes<br>2. 30minutes- 1hour<br>3. $\geq$1hour

Part IV:- Client satisfaction level with family planning services						
		**Strongly disagree**	**Disagree**	**Neutral**	**Agree**	**Strongly agree**
1.	I was provided with clear and comprehensive information about family planning methods.					
2.	The healthcare provider used language that was easy for me to understand.					
3.	I felt comfortable asking questions during the consultation.					
4.	I was informed about what to do in case of side effects or problems.					
5.	I was given clear instructions on when to return for follow-up visits.					
6.	The healthcare provider treated me with respect and dignity.					
7.	My cultural and personal beliefs were acknowledged and respected during the consultation.					
8.	I felt my choices were valued and supported by the healthcare provider.					
9.	The facility was clean and organized.					
10.	Educational materials about family planning were available and informative.					
11.	The waiting time to receive services was reasonable.					
12.	I am satisfied with the quality of care I received.					
13.	I feel confident contacting this facility if I have questions or concerns.					
14.	I would return to this facility for family planning services in the future.					
15.	I would recommend this facility to friends and family.					

Interview Guide: Exploring client satisfaction with family planning services

General introduction for the facilitator

Hi, how are you? Are you comfortable for discussion?

I am …………………. (Full name of the facilitator), and I am going to facilitate our discussion on your personal views and experiences regarding family planning services and how they have impacted your life. Before we go further, let us introduce ourselves.

Interview questions

1) Can you describe your overall experience with family planning services?

Probing questions:

- What motivated you to seek family planning services?
- What challenges did you face in accessing these services?
- How did the healthcare providers interact with you during your visit?

2) How do cultural, social, or religious factors influence your decisions about family planning?

Probing questions:

- Were there any cultural or family pressures that influenced your decisions?
- How do you feel your cultural or religious beliefs were considered during your consultation?
- Did you experience any stigma or judgment related to using family planning methods?

3) What misconceptions or fears do you have about family planning?

Probing questions:

- Did you have any concerns or misconceptions about family planning methods before coming here?

- Were your questions or concerns addressed during counseling?
- How confident were you in the information provided about method effectiveness?

4) Have you or others you know experienced issues with contraceptive effectiveness?

Probing questions:

- Have you ever experienced method failure, such as an unintended pregnancy?
- How did this experience affect your trust in family planning methods?
- What emotional impact did it have on you or your family?

5) How satisfied are you with the family planning services provided?

Probing questions:

- Were the family planning methods you wanted available?
- Were the facilities clean and organized?
- Did you feel that your privacy was maintained during consultations?

6) What support or follow-up were you provided with after receiving family planning services?

Probing questions:

- Were you informed about potential side effects and how to manage them?
- Did the provider encourage you to return for follow-up care?
- Do you feel confident contacting the facility if you have concerns or questions?

7) How do family planning services impact your overall quality of life?

Probing questions:

- How do you feel about your ability to make reproductive health decisions after receiving services?
- Has the service improved your confidence or reduced stress related to family planning?

- What role does your partner or family play in supporting your decisions?

8) What aspects of the service did you find most or least satisfying?

Probing questions:

- What did you like most about the services provided?
- What challenges or barriers do you think need improvement?
- If you could change one thing about the service, what would it be?

Thank you for sharing your experiences. Is there anything else you would like to add or clarify about your experience with family planning services?
